# Comparisons between the Neighboring States of Amazonas and Pará in Brazil in the Second Wave of COVID-19 Outbreak and a Possible Role of Early Ambulatory Treatment

**DOI:** 10.3390/ijerph18073371

**Published:** 2021-03-24

**Authors:** Francisco G. Emmerich

**Affiliations:** Federal University of Espirito Santo, Campus de Goiabeiras, Vitoria-ES 29075-910, Brazil; fgemmerich@terra.com.br

**Keywords:** SARS-CoV-2, COVID-19, mortality, treatment, comparison, statistics, Brazil

## Abstract

Brazil and many countries are now experiencing a second wave of the COVID-19 outbreak. The objective of this study is to compare results with statistical samples involving millions of people in the two largest neighboring states in Brazil, Amazonas and Pará, which in the first wave were similar but now show significant different results in combating COVID-19. During the first wave, in May 2020, the maximums of the 7-day average daily deaths per population of Amazonas and Pará were similar: 15.7 and 17.1 deaths per day per million people, respectively, which means a ratio 15.7/17.1 = 0.92 ≈ 1. Now, in the second wave of COVID-19 outbreak, Amazonas has entered a serious situation; meanwhile, Pará has presented a much smaller growth in the mortality. The accumulated mortality per population from 11 November 2020 to 15 March 2021 of Amazonas and Pará are 1645 and 296 deaths per million people, respectively. As 1645/296 = 5.55, Amazonas is presenting an accumulated mortality per population more than five times that of Pará. Future in-depth research can provide a grounded answer to explain this significant difference, nonetheless the explicit support of the Pará state government, after 21 May 2020, to early ambulatory treatment may have played some role on this result.

## 1. Introduction

The COVID-19 pandemic has been impacting the world since the beginning of 2020 [1,2] and two waves of COVID-19 outbreak [3] have hit many countries. As pointed out by Jindal et al. [4], according to the Centers for Disease Control and Prevention of the United States of America (USA), there are two ways to control the damage of a viral infection: (1) reduce the spread of the virus and, (2) decrease the associated disease severity. Concerning item (1), most countries issued complete or partial lockdown in many cities and measures of prevention such as social distancing, wearing a mask, washing hands, post-exposure prophylaxis, and staying at home quarantined under signals of infection [1,2,4,5]. Concerning item (2), some medicines such as hydroxychloroquine, azithromycin, ivermectin and others were proposed and tested against the SARS-CoV-2 virus in some stages of the disease [6,7,8,9], and several vaccines were developed in record time [10,11].

Some groups of researchers showed the advantages of early ambulatory treatments for COVID-19 [7,8,12,13,14], but, as commented on by Paul [15], the differences between patients given treatment for COVID-19 or not require exceptionally large sample sizes for appropriate adjustment. There are many recruitment difficulties [16] in conducting experimental studies involving large number of patients. Although studies with 10,000–20,000 patients or more are important, the scope and the objective of this research note is not to discuss them but to work on comparisons of statistical samples involving millions of people in two states of a country in the second wave of COVID-19 outbreak. This country is Brazil, which occupies a vast area (about half of South America) with a relatively large population, which corresponds to about 45% of the population of South America, 30% of Europe and 65% of the USA. Therefore, cross-comparison for Brazil can be relevant and the information provided can be extrapolated to the world in many cases.

As pointed out by Pearce et al. [17], comparisons are important because, despite some difficulties, it is possible to learn a great deal from comparing countries, states, and regions, and they can play a major role in our learning what works best for controlling COVID-19. The number of options is not high, and some alternatives to comparisons, such as randomize a lockdown or other aspects of physical distancing, are impossible or unethical [17]. There could be trials of intensive population testing, or prophylactic treatment of household contacts, but few have been launched, and the clock is ticking accumulating more deaths [17].

Comparisons between countries of Africa, Asia, Europe, Central, North and South America, and Oceania were performed [18,19,20,21,22,23,24,25,26,27,28]. Most of these studies analyzed the diverse strategies in combating the disease and report parameters involving the number of cases and the mortality. Some of the studies involved themes such as testing coverage [23], economic valuation [24], response strategies [25], age distribution [26,27], and seasonal climate changes [28], among others.

Comparisons involving states and country regions were also presented [29,30,31,32,33]. For example, Rath et al. [29] studied selected states of India by analyzing parameters such as case fatality rate and population density. La Gatta et al. [30] used graph-based machine learning to compare the forecasts of the trained model with available data about the Covid-19 epidemic spread in different regions of Italy. Cavalcante et al. [32] described the evolution of the pandemic until 16 May 2020 in Brazil, analyzing the number of cases and mortality, making comparisons between states, regions, and also with other nine countries. Orellana et al. [33] studied the excess overall mortality until 19 May 2020 in the state of Amazonas (Brazil), focusing mainly on its capital, analyzing parameters such as age bracket, sex, place of death, epidemiological week, and specific causes of death. These authors [33] made comparisons with the pre-COVID-19 mortality in 2018–2019 and presented comments about other Brazilian states and the world.

Most of the mentioned comparisons studies [18,19,20,21,22,23,24,25,26,27,28,29,30,31] involve the first wave of COVID-19 outbreaks, and two of them were focused more specifically on Brazil and its states [32,33] analyzing results until 16–19 May 2020. The objective of the present work is to make comparisons between the neighboring states of Amazonas and Pará in Brazil in the second wave of the COVID-19 outbreak. This is particularly relevant because in the first wave of the COVID-19 outbreak, the maximums of the mortality rate in the states of Amazonas and Pará were quite similar considering their populations, but now, in the second wave, there are significant differences in the results. To the author’s knowledge, the present work is one of the first comparison studies between two neighboring states that were quite similar during the maximum of the first wave, and now, in the second wave, are presenting significant different results in combating COVID-19.

**Figure 1:**
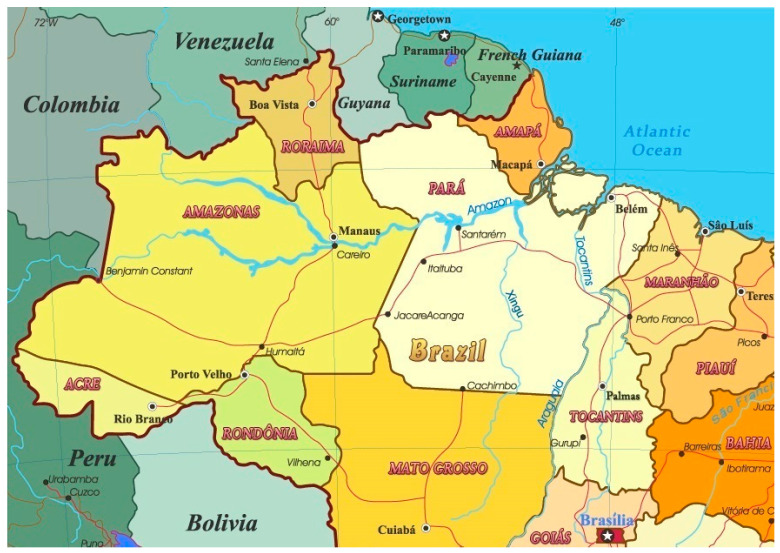
Map of the states of Amazonas and Pará in Brazil and their neighboring states and international borders. Reproduced from http://www.geographicguide.net/america/brazil-map.htm (accessed on 15 February 2021)—Adapted with permission from copyright © Geographic Guide—World in Pictures.

Amazonas and Pará are the two largest states of Brazil by area, located in the northern region of the country and traversed by the Amazon River (cf. Figure 1). Their summed area corresponds to 33% of the country (Amazonas: 18.3% and Pará: 14.6%), and their populations are 4.208 and 8.691 million people, which correspond respectively to 2.0% and 4.1% of the total Brazilian population (211.8 million people). Although the population of Amazonas is 0.48 (about half) of Pará, the metropolitan regions of their state capitals (Manaus-AM and Belém-PA) have similar populations: Greater Manaus (13 municipalities): 2.72 million people and Greater Belém (7 municipalities): 2.51 million people. Excluding the two large metropolitan regions surrounding their capitals, the other municipalities, 49 in Amazonas and 137 in Pará, have average populations of 30 and 45 thousand people, respectively. Furthermore, among other factors, the two states are relatively similar in climate, in the public healthcare system, in the socioeconomic status (SES) distribution, in the level of education of the population, and in the age and gender composition [34,35]; the Human Development Index (HDI) of 2010 for Amazonas, Pará and Brazil as a whole are, respectively, 0.674, 0.646 and 0.699. These inherent similarities of the two states, together with the specific circumstances during the first and in the second wave of COVID-19 outbreak, will evidence that the comparisons are appropriate and helpful. Looking for the main differences of behavior between the two states in the second wave may serve as an example for other states or other geographic entities in the combat of the COVID-19 pandemic.

An important starting point for the present work is to define what is the most appropriate parameter to use in the comparisons. The number of cases per million people is a rate that is frequently reported for comparison purposes, but the testing practice of different countries and states to identify cases may vary [24,36,37]. Other parameters, such as infection fatality ratio and case fatality ratio are other relevant measures, but they depend directly on the testing practices to identify cases, which usually are not uniform and vary. In the case of Brazil, the testing practice to identify COVID-19 cases varies from state to state and also between municipalities in the same state, so using a parameter such as the case fatality ratio to compare states may not be very appropriate.

Although deaths per million people is a crude rate, many consider it a useful comparator [24,36,37]. As pointed out by Fitzpatrick [37], although some COVID-19 reported deaths include just those tested positive, which is mainly the case in hospitalized patients; other reported deaths include those where COVID-19 is regarded highly likely, without confirmation. Despite the possibility of doubt in some circumstances, in the case of Brazil, the number of deaths by COVID-19 per million people is the parameter that may be more appropriate for comparisons between states and municipalities, because the statistics of the population is well performed by a federal institute (Instituto Brasileiro de Geografia e Estatística—IBGE) since 1938, and, according to a federal regulation (Art. 77 of Law No. 6015 of 15 December 1973), the death is an event in which a physician is responsible for certifying the cause, and a death certificate must be issued in a civil registration office, before burial. Only in cases where there is no physician, two qualified persons who have witnessed or verified the death can inform the cause. However, the great majority of the municipalities in Brazil have physicians, which are paid for with the support of the municipalities, the states, and the Brazilian Unified Health System (Sistema Único de Saúde—SUS). Despite the possibility of doubt in some cases of highly likely COVID-19 deaths, without confirmation, it is probably that these cases in Brazil may be distributed evenly among the municipalities and states of the regions, and not concentrated in certain municipalities and states (of the region). Therefore, taking into account all these reasons, it is appropriate to assume that the municipalities inside the regions of Brazil have reasonable uniformity in the criteria for the notification of the COVID-19 deaths of the patients. In addition, it is worth comment that the notifications from the municipalities are what generate the data for the states and for the country, which appear daily in the released statistics. All things considered, the number of deaths per million people is the parameter that will be used in the comparisons of the present work.

## 2. Materials and Methods

The daily deaths of COVID-19 in Brazil and other statistical data of the pandemic are provided by the municipalities and the states, and compiled by the Ministry of Health, which provides a spreadsheet in a CSV format available on its website (https://covid.saude.gov.br/ (accessed on 15 March 2021)). The File S1 used here was obtained on 15 March 2021, and is reproduced in the Supplementary Materials. It involves daily data since 25 February 2020 of the country, of the 27 federation units (26 states and one federal district), and of the 5570 municipalities.

As the present work involves the country and the federation units, it was sufficient to take the first part of the above spreadsheet and export the content of this primary data to the File S2 of three tabs created by the author, which is available openly in the Supplementary Materials for those who are interested. This spreadsheet contains the primary data, the determination of the useful parameters, and the data used to make the graphs. In the part of primary data, the columns of interest in the present study are region, state, date, population, accumulated deaths, and daily deaths. Other columns, such as: accumulated cases, new cases, new recovered cases, and follow-up new cases, were not used here, but may be of interest for other works. The averages of deaths per day were calculated on a 7-day basis, by taking the data of the considered day and the six previous days. The details of how the data were worked out in this spreadsheet are presented in Appendix A in the first three paragraphs.

The graphs were made in the Excel File S3 of 28 tabs, which is also available openly in the Supplementary Materials for those interested. This spreadsheet provides graphs of the country and all 27 federation units in a suitable resolution. Appendix A, in its fourth paragraph, provides details of how the data were worked out in the spreadsheet. The figures with the graphs shown in this work can be compared with graphs, normally of lower resolution, provided daily by the Brazilian press through the “Consórcio de Veículos de Imprensa”. For example, the webpages of Uol and Globo, which present daily statistical graphs of COVID-19 for the country and the federation units, are indicated in the references [38,39].

## 3. Results and Discussion

**Figure 2:**
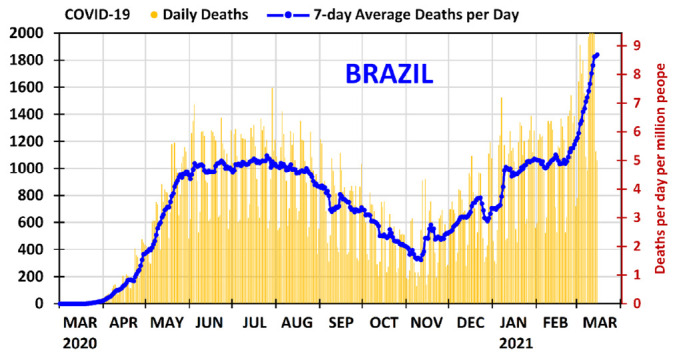
Daily deaths and 7-day average deaths per day of COVID-19 in Brazil from 1 March 2020 to 15 March 2021. The deaths per day per million people can be found using the secondary vertical axis on the right.

As shown in Figure 2, Brazil suffered the first wave of COVID-19 outbreak between March and 10 November 2020. The period of highest mortality of the first wave occurred between the end of May 2020 and the beginning of September 2020, with the maximum of the 7-day average daily deaths occurring on 25 July 2020: 1097 deaths per day, which corresponds to 5.1 deaths per day per million people. The second wave of the pandemic began on 11 November 2020 and is affecting most states. It is worrying because the mortality rate now, in March 2021, is significantly higher than that of the maximum of the first wave, and it is mainly related to new, more contagious, variants of the virus [11,40], and also to reductions in the measures of prevention. In February 2021, the 7-day average daily deaths was 1000–1200 deaths per day (4.7–5.7 deaths per day per million people). In March 2021, the mortality rate is increasing steadily; on 15 March 2021, the 7-day average daily deaths has reached 1841 deaths per day, the highest in the world at the moment (in absolute value), which corresponds to 8.7 deaths per day per million people.

**Figure 3:**
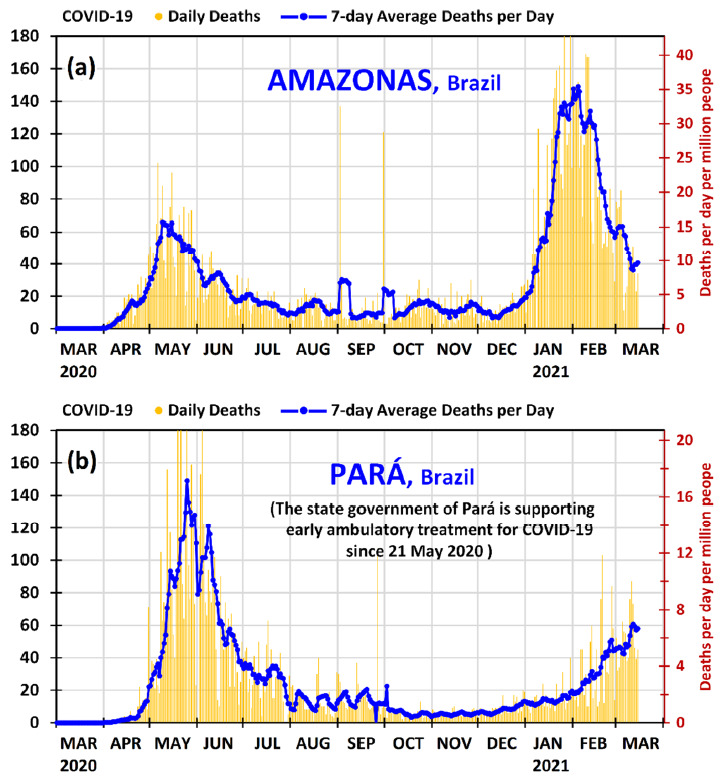
Daily deaths and 7-day average deaths per day of COVID-19 in the states of (**a**) Amazonas and (**b**) Pará in Brazil from 1 March 2020 to 15 March 2021. The deaths per day per million people can be found using the secondary vertical axis on the right.

As shown in Figure 3, during the month of May 2020, Amazonas and Pará experienced strong first waves of COVID-19 outbreak. The maximums of the 7-day average daily deaths reached 66 deaths per day in Amazonas on 9 May 2020 and 149 deaths per day in Pará on 25 May 2020, which correspond to 15.7 and 17.1 deaths per day per million people, respectively. The difference in the daily deaths per population was only −9%, and therefore the two states were quite similar because the ratio 15.7/17.1 = 0.92 ≈ 1.

In that situation, in March–May 2020, each state and the municipalities separately adopted measures to contain the pandemic, because in Brazil the Supreme Federal Court has decided that the municipalities, the states and the federal government have autonomy of action, in their respective jurisdictions, to combat COVID-19 [41]. In general, the mayors and governors implemented partial lockdown in some cities and suggested measures of prevention such as those mentioned in Section 1. However, the government of the state of Pará had a different additional attitude because: (1) a private healthcare plan operator (Unimed Belém) was with successful results with early ambulatory treatment dispensing medicines such as those mentioned in Section 1 to their patients upon prescription from their physicians [42]; (2) some municipalities, such as Afuá, were already acquiring such medicines [43]; and (3) the municipality of Ourilândia do Norte (Center-South of Pará, 33.1 thousand people and HDI of 2010 = 0.624) was also with successful results with early ambulatory treatment dispensing medicines such as those mentioned in Section 1 to patients upon prescription from their physicians at its municipal public healthcare unit [44]. On 21 May 2020, the state government of Pará, as documented on the official state agency [45] and in the press [46], acquired hundreds of thousands of capsules of medicines for covid-19 to distribute to the municipalities in the state for use by people with symptoms or a confirmed diagnostic of COVID-19. On that occasion it was informed that the effectiveness of early ambulatory treatment for COVID-19 was not yet scientifically proven and that the medicines could only be prescribed by physicians if they so wish; it was informed that the main role of the state government of Pará was to guarantee the supply of the medicines. A concise complementary discussion of this issue is left at the end of this section, supplemented with Appendix B.

As demonstrated in Figure 3, both states managed to control the first wave of COVID-19 outbreak; however, Pará presented a much faster reduction after the maximum of the first wave, not only in relation to the state of Amazonas, but in relation to all states in Brazil. After the first wave maximum, the state of Pará reduced the 7-day average daily deaths by 83% in 46 days (from 25 May 2020 to 10 July 2020) and by 95% in 70 days (from 25 May 2020 to 3 August 2020), which is a remarkable result.

Some points can be emphasized in this matter: (1) The physicians of the public healthcare system in the state of Pará started to know in May 2020 that the proposed early ambulatory treatment was working well for a private healthcare plan operator [42] and for a municipality [44]; (2) the state government of Pará, which have made early ambulatory treatment feasible by purchasing the medicines, has a great power of influence; and, importantly, (3) the patients and the municipalities did not have to purchase and pay for the medicines—the state government of Pará did so. Consequently, it is likely that the result obtained in Pará (the best in Brazil) in decreasing at the shortest time the maximum of the death rates of the first wave of COVID-19 outbreak may be due to the explicit support of the state government and the adhering of the municipalities and their physicians in the public healthcare system to the early ambulatory treatment. The role of early ambulatory treatment in the second wave is discussed latter.

As can be observed in Figure 3, the second wave of the COVID-19 outbreak has hit the state of Amazonas severely. From the middle of December 2020, Amazonas presented an increase in the daily deaths, which grew significantly in January 2021. The 7-day average daily deaths in Amazonas remained above 120 deaths per day (15.3 deaths per day per million people) from 18 January 2021 to 15 February 2021, and then decreased to 60 deaths per day on 27 February 2021 and then to 40 deaths per day on 11 March 2021. Meanwhile, its neighboring state, Pará, which had a first wave as strong as that of Amazonas in May 2020, taking into account the populations, has been in a different situation. In the second wave Pará has presented a much smaller growth than Amazonas. The 7-day average daily deaths in Pará increased from 13 to 20 deaths per day from 1 to 31 January 2021, from 18 to 45 deaths per day from 1 to 28 February 2021, and from 46 to 58 deaths per day from 1 to 15 March 2021. The latter value corresponds to 6.7 deaths per day per million people. Despite the fact that Pará is in a situation where the maximum of the second wave is not yet defined as in Amazonas, it may be useful to make a comparison with the current situation. In the second wave (until 15 March 2021), the maximums of the 7-day averages of daily deaths were 149 deaths per day in Amazonas on 4 February 2021 and 61 deaths per day in Pará on 12 March 2021, which correspond to 35.4 and 7.0 deaths per day per million people, respectively. Since 35.4/7.0 = 5.1 ≈ 5, the ratio of the maximums of the 7-day average daily deaths per population between Amazonas and Pará has increased from about 1 to 5 from the first wave to the second wave.

It is worth commenting on the fact that the reduction of the daily deaths in Amazonas from the middle of February 2021 is related to several measures of prevention and partial lockdown in some cities that were issued from January–February 2021; mainly in Manaus and its metropolitan area. Moreover, the vaccination program in Brazil, which started on 17 January 2021, is also contributing to part of the decrease, because, due to the circumstances, the state of Amazonas has received priority: 8.07% of its population have received the first dose by 14 March 2021 (almost the double of the Brazil average, 4.59%) [47,48]. Older people, people with disabilities in care institutions, and health professionals have been included in the priority group, and over 60% of indigenous people above the age of 18 have received the first dose by 26 February 2021 [49]; and the indigenous of Amazonas are receiving a higher priority [50].

**Figure 4:**
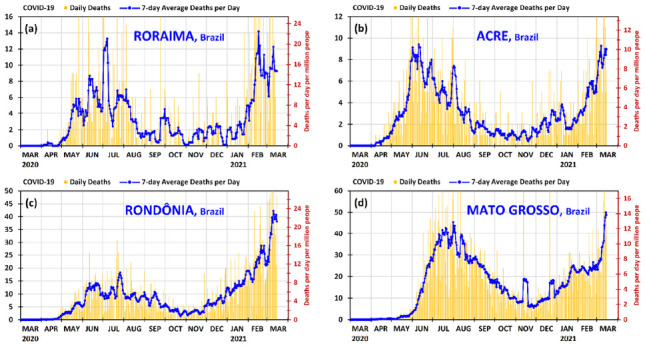
Daily deaths and 7-day average deaths per day of COVID-19 in the states of (**a**) Roraima, (**b**) Acre, (**c**) Rondônia and (**d**) Mato Grosso in Brazil from 1 March 2020 to 15 March 2021. The deaths per day per million people can be found using the secondary vertical axis on the right.

Since Amazonas has entered in a difficult situation during the second wave, and the graph of the state of Pará has already been shown, it is instructive to observe the panorama of the other neighboring states of Amazonas (Roraima, Acre, Rondônia, and Mato Grosso). Figure 4 provides the graphs of daily deaths and 7-day average deaths per day of Roraima, Acre, Rondônia, and Mato Grosso from 1 March 2020 to 15 March 2021.

Positively, new, more contagious, variants of the virus COVID-19 have been contributing to the situation in Amazonas and many states of Brazil in the second wave. Although two variants have been verified in Brazil [40], P.1 (B.1.1.28) and VOC 202012/01 (B.1.1.7), the variant P.1 is the predominant [11,40]. In all likelihood, it is not a matter of timing that the new variants in the second wave may reach Pará with the intensity that Amazonas has been reached, since the second wave has already reached Brazil with severity (cf. Figure 2), and most states are presenting a high increase in daily deaths. In the case of Pará, the larger increases in the 7-day averages of daily deaths have occurred from 18 to 26 February 2021 and from 6 to 12 March 2021, but these increases are not as high as those of Amazonas from 4 to 25 January 2021. Moreover, as shown in Figure 4, all the other neighboring states of Amazonas (Roraima, Acre, Rondônia and Mato Grosso) are presenting accumulated mortality per population much larger than that of Pará, as will be detailed in Table 1. As shown in Figure 4, the situation is serious because these neighboring states of Amazonas are presenting high daily deaths; therefore, it is highly likely that the variant P.1 is one of the main causes of these significant increases in the daily deaths because it is the predominant variant in Brazil [11,40]. On 15 March 2021 the 7-day average daily deaths in the states of Roraima, Acre, Rondônia and Mato Grosso were 9.3, 9.0, 38.1 and 48.7 deaths per day, which corresponds, respectively, to 14.7, 10.1, 21.2 and 13.8 deaths per day per million people. These values are, respectively, 120%, 51%, 318% and 107% higher than that of Pará.

To better compare Amazonas and Pará with the country and other federation units in the second wave of COVID-19 outbreak, some parameters of interest are provided in Table 1 for all states, the federal district, and the country as a whole. The last four columns of Table 1 provide the accumulated mortality per population in the second wave (from 11 November 2020 to 15 March 2021)—in absolute values and in relation to the state of Pará; and the ratio between the maximums of the 7-day average daily deaths in the second wave and in the first wave (from 1 Mar 2020 to 10 November 2021)—in absolute values and in relation to the state of Pará. It is worth commenting on that the data of these comparisons, and those of other comparisons such as the ratio between the accumulated mortality per population in the second wave and in the first wave, can be found in File S2 of Supplementary Materials. Because the second wave of the pandemic is still taking place, the accumulated mortality increases every day, especially for the various states where the average daily deaths is high or continues to increase. The last two columns, which depend on the maximum of the 7-day average daily deaths in the second wave, may also vary in cases where the maximum has not yet been reached.

The antepenultimate and the last columns of Table 1 demonstrate quantitatively that the state of Pará is the best of the North region (where the virus variant P.1 was first found in Brazil [11,40]) in the fight against COVID-19. Moreover, Pará, even located in the presently risky North region, has performed best against COVID-19 when compared with most states of the other regions, and with the country in general. Amazonas, Pará, and Brazil (as a whole) have presented (until 15 March 2021) an accumulated mortality per population in the second wave of 1645, 296, and 550 deaths per million people, respectively; this means that Amazonas and the country (as a whole) are presenting values that are 5.55 and 1.86 times that of Pará.

**Figure 5:**
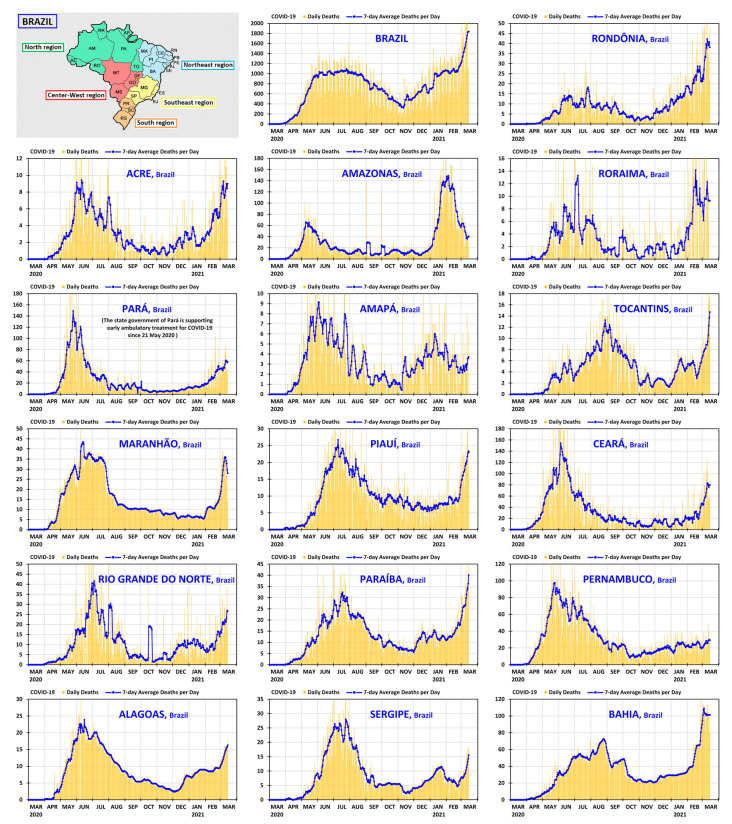
Daily deaths and 7-day average deaths per day of COVID-19 in Brazil and in the states of the North and Northeast regions of Brazil from 1 March 2020 to 15 March 2021.

**Figure 6:**
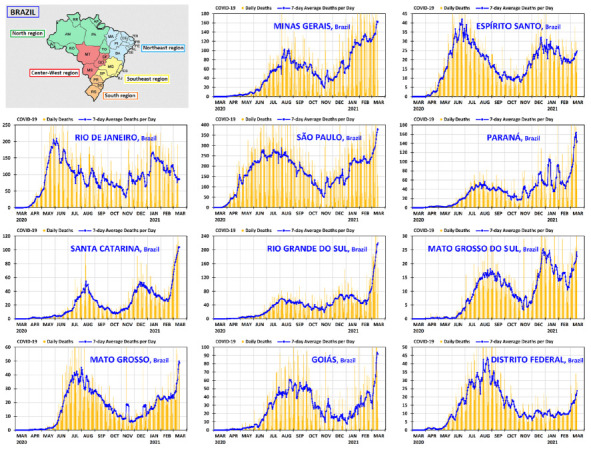
Daily deaths and 7-day average deaths per day of COVID-19 in the states of the Southeast, South and Center-West regions of Brazil from 1 March 2020 to 15 March 2021.

It is instructive to complement the prospects offered by Figure 3 and Figure 4 with a unified panoramic view of the country, region by region. The graphs of daily deaths and 7-day average deaths per day—from 1 March 2020 to 15 March 2021—are presented in Figure 5 and Figure 6 for all states of Brazil and its federal district, grouped by region, following the same order as in Table 1. The North and Northeast regions are shown in Figure 5, and the Southeast, South and Center-West regions in Figure 6. In both figures, there is a small map showing the positions of the states and regions. The country graph is shown at the beginning of Figure 6, so that all relevant graphs can be seen together in the same resolution.

Although it is necessary to perform future in-depth research to provide a more grounded answer to explain with clarity the significant difference of the good result of Pará in combating COVID-19 in relation to that of Amazonas in the second wave (mainly in January–February 2021, when the virus variant P.1 was already present in the North region and in some states of Brazil), it is likely that the strong support of the Pará state government, after 21 May 2020, for early ambulatory treatment, may have played some role in the good result. This inference is being made because the two neighboring states were similar in the relative mortality rates at the maximums of first wave, and in the second wave they have presented significant differences, and the main difference of the behavior of the government of the two states, after 21 May 2020, was the strong support of the Pará state government to early ambulatory treatment.

It is worth commenting that since the municipalities in Brazil have autonomy in their jurisdictions to combat COVID-19, some of them have supported early ambulatory treatment policies, while others have not. Hence, it is difficult to generalize saying that a given state, as a whole, has implemented early ambulatory treatment or not. For example, in the state of Maranhão, the Regional Council of Medicine supported early ambulatory treatment from the beginning of May 2020 [51] with the medicines mentioned in Section 1, but the state government of Maranhão did not support early ambulatory treatment. In contrast to other states, in Pará there is a clear message and action of the state government that was passed to the population and to the municipalities. Most of the municipalities and the physicians of the public healthcare system in the state of Pará adhered to early ambulatory treatment after the difficult situation in May 2020, as it is detailed in Appendix B, which also discusses why this was not followed in the state of Amazonas.

As said previously in the discussion of the first wave, the measures of prevention and lock downs in April–May 2020 helped the state of Amazonas to reduce the mortality at that time. However, relaxations in measures of prevention and the more contagious P.1 variant have caused the increase in the mortality in Amazonas from December 2020, and especially from the middle of January 2021, with daily deaths higher than in May 2020. This situation (with the more contagious virus variant P.1) spread to the neighboring states of Amazonas, but Pará was the neighboring state that has suffered the least, as shown in Figure 4 and Table 1. This demonstrates that is very likely that the early ambulatory treatment implemented in the public healthcare system in the state of Pará may be one of the main causes for its relatively good results in the second wave.

Because the virus is still causing high mortality in most states of Brazil, which intensified in March 2021, as can be directly observed in Figure 5 and Figure 6, measures of prevention [1,2,4,5] such as social distancing and wearing a mask, among others, must continue to be used; partial lockdown in some cities must also be issued, depending on the circumstances. The vaccines that were developed for COVID-19 in record time [10,11] are a major advance for humanity and for the science, technology, and innovation of the 21th century, and must be strongly supported by everyone. However, vaccination will not be so fast, especially in countries with large populations and less technological development, because most of them have not invested in advanced purchase of a significant number of doses. An additional difficulty is that in the second half of January 2021 the European Union and the United States of America—two major blocks of vaccine producers—announced some measures to restrict the export of vaccines [52,53]. The vaccination program in Brazil started in middle January 2021, and by 14 March 2021 4.59% of the population have received the first dose [47,48] (either Coronavac or Oxford/AstraZeneca). Two important research/development centers of Brazil (Instituto Butantan and Fiocruz) are producing the vaccines by using ingredients from China and India. The federal government and some state governments are negotiating with other producers; there is progress but there are also some difficulties to obtain large amounts of vaccine doses, mainly in the next months. Therefore, early ambulatory treatment for Covid-19 may still be important as shown by some groups of researchers [7,8,12,13,14]. The goal should always be to save as many lives as possible. The author considers that depending on the specific circumstances, similar comparisons can be made in other geographically neighboring regions, involving countries, states, and municipalities.

## 4. Concluding Remarks

Amazonas and Pará, the two largest neighboring states in Brazil, were in a similar situation during the worse period of the first wave of COVID-19 outbreak in May 2020, and each state and the municipalities separately adopted procedures to contain the first wave of COVID-19 outbreak, such as partial lockdown in some cities and used several measures of prevention. However, the Pará state government, after 21 May 2020, started a strong support to early ambulatory treatment in the public healthcare system. The outcome was that Pará presented the faster reduction in Brazil of daily deaths after the maximum of the first wave: the 7-day average deaths per day decreased 95% in 70 days (from 25 May 2020 to 3 August 2020).

Now, in the second wave of COVID-19 outbreak, the state of Amazonas faced a serious situation, mainly from the middle of January to the middle of February 2021; meanwhile, the state of Pará has presented a much smaller growth in the death rates, presenting an accumulated mortality during the second wave much smaller than that of Amazonas, the other neighboring states of Amazonas, and also most of the other states of Brazil. The accumulated mortality per population so far in the second wave of COVID-19 outbreak, (from 11 November 2020 to 15 March 2021) of Amazonas and Pará are 1645 and 296 deaths per million people, respectively. This means that Amazonas is presenting an accumulated mortality per population in the second wave more than five times that of Pará, which is a significant difference. Fortunately, this large difference is decreasing because Amazonas has implemented several measures of prevention (mainly from January 2021); the vaccination program, which started in late January 2021, is also slightly contributing to decrease the daily deaths.

Although it is necessary to have future in-depth research to provide a grounded answer to explain with clarity this significant difference between Amazonas and Pará in the second wave of COVID-19 outbreak, mainly in January–February 2021 (when the virus variant P.1 was already present in the North region and in some states of Brazil), it is likely that the strong support of the Pará state government, after 21 May 2020, to early ambulatory treatment, and the adhering of the municipalities and their physicians in the public healthcare system to early ambulatory treatment, may have played a role in the good result of Pará for controlling COVID-19 (even with the presence of the virus variant P.1) in comparison with the states of the region and many states of Brazil. The comparisons offered in the present work indicates that early ambulatory treatment is an option that should not be a priori neglected in the public healthcare policies to combat COVID-19.

Depending on the specific circumstances, the type of comparison presented here can be applied, in a similar way, in other neighboring geographic entities, such as countries, states, regions, and municipalities.

## Figures and Tables

**Table 1:** Accumulated mortalities per population in the second wave of COVID-19 outbreak (from 11 November 2020 to 15 March 2021); and the ratios between the maximums of the 7-day average daily deaths in the second wave and in the first wave (from 1 March 2020 to 10 November 2021)—for all states of Brazil—in absolute values and in relation to the state of Pará.

State	Abbr.	Region	Population	Accumulated Mortality Per Population in the Sec. Wave (Deaths Per Million People)	Accumulated Mortality Per Population in the Sec. Wave (in Relation to the State of Pará)	Max. 7-Day Av. Deaths Per Day Sec. Wave/ First Wave	Max. 7-Day Av. Deaths Per Day Sec. Wave/ First Wave (in Relation to the State of Pará)
Rondônia	RO	North	1,796,460	1076	3.63	2.31	5.68
Acre	AC	North	894,470	475	1.60	0.98	2.42
Amazonas	AM	North	4,207,714	1645	5.55	2.26	5.55
Roraima	RR	North	631,181	851	2.87	1.06	2.61
Pará	PA	North	8,690,745	296	1.00	0.41	1.00
Amapá	AP	North	861,773	489	1.65	0.66	1.61
Tocantins	TO	North	1,590,248	366	1.24	1.11	2.72
Maranhão	MA	Northeast	7,114,598	192	0.65	0.83	2.03
Piauí	PI	Northeast	3,281,480	355	1.20	0.87	2.14
Ceará	CE	Northeast	9,187,103	315	1.06	0.54	1.32
Rio Grande do Norte	RN	Northeast	3,534,165	376	1.27	0.65	1.59
Paraíba	PB	Northeast	4,039,277	452	1.53	1.24	3.04
Pernambuco	PE	Northeast	9,616,621	275	0.93	0.31	0.75
Alagoas	AL	Northeast	3,351,543	280	0.94	0.68	1.68
Sergipe	SE	Northeast	2,318,822	386	1.30	0.56	1.36
Bahia	BA	Northeast	14,930,634	369	1.24	1.50	3.68
Minas Gerais	MG	Southeast	21,292,666	539	1.82	1.59	3.91
Espírito Santo	ES	Southeast	4,064,052	687	2.32	0.77	1.88
Rio de Janeiro	RJ	Southeast	17,366,189	773	2.61	0.80	1.96
São Paulo	SP	Southeast	46,289,333	529	1.79	1.31	3.22
Paraná	PR	South	11,516,840	706	2.38	2.91	7.13
Santa Catarina	SC	South	7,252,502	765	2.58	1.90	4.67
Rio Grande do Sul	RS	South	11,422,973	797	2.69	3.68	9.04
Mato Grosso do Sul	MS	Center-West	2,809,394	708	2.39	1.40	3.44
Mato Grosso	MT	Center-West	3,526,220	689	2.33	1.10	2.70
Goiás	GO	Center-West	7,113,540	522	1.76	1.54	3.77
Distrito Federal	DF	Center-West	3,055,149	448	1.51	0.54	1.33
							
BRAZIL	BRA		211,755,692	550	1.86	1.68	4.12

## Data Availability

The File S1 of Supplementary Materials was obtained online at https://covid.saude.gov.br/ (accessed on 15 March 2021). Each day the spreadsheet is updated, keeping the data of the past dates. The Files S2 and S3 of Supplementary Materials were made by the author as explained in Appendix A.

## Supplementary Materials

The following are available online at https://www.mdpi.com/1660-4601/18/7/3371/s1, File S1: provides the daily deaths and other statistical data of COVID-19 in Brazil from 25 February 2020 to 15 March 2021. File S2: contains the primary data, the determination of useful parameters, and the data used to make the graphs. File S3: provides the graphs of the country and of the federation units of Brazil.

## Appendix A. Details on the Spreadsheets with the Data/Parameters and the Graphs

As the complete CSV File S1 is exceedingly long, mainly because it involves daily data of 5570 municipalities, it is convenient to use a TXT file editor to separate the suitable part of the data that is the focus of the analysis, before opening this part in a spreadsheet editor. As the present work involves only the country, the states and the federal district, and not the municipalities, it was sufficient to take the first 10,783 lines.

The primary data contained in the above lines were exported to the Excel File S2 of three tabs in the tab “CO-VIDBR_15mar2021-data-calc”. The primary data is spread over an area of 17 columns and 10,781 rows kept intact (from column A to Q). Two rows (306 and 327) of the original spreadsheet were removed, because they were repeating the date of the previous row and the number of new deaths were zero in the original rows (306 and 327). Column R was left blank, and columns S, T, and U were used to place the necessary data of the graphs. Columns S and T provide the date and daily deaths and are copies of columns H and N. Column U provides the 7-day average daily deaths, which was calculated as 1/7 of the sum of daily deaths between the day considered and the previous six days. As there are no deaths before 17 March 2020, the 7-day average daily deaths from 1 March 2020 to 6 March 2020 was taken equal to zero in column U. From 7 March 2020 to the final date (15 March 2021) it was used the formula Un = (SUM(T(n − 6):Tn))/7, where n is the number of the row. For example, for row 13 (7 March 2020) it was used U13 = (SUM(T7:T13))/7. The number of rows in columns S, T and U for the graph of each federation unit is equal to the number of days between 1 March 2020 and 15 March 2021. For the country and each federation unit, the row corresponding to the initial date of the graphs (1 March 2020) was used, from column V to AK, to place the calculations of several parameters of interest, such as the accumulated mortality in each wave of the pandemic. All mathematical operations are simple (addition, subtraction, multiplication, and division) and can be seen in the formulas openly disclosed in the spreadsheet.

Two additional tabs (“Parameters-pop-2019” and “Parameters-pop-2020-final”) were created to compile the results related with the parameters. The Parameters-pop-2019 tab was obtained by copying the data of the first tab from column V to AK and from row 7 to 10,781, and pasting the data as “values”. As the spreadsheet with primary data from the Ministry of Health provides population data updated in 2019, an additional tab, Parameters-pop-2020-final, was copied from the previous one, replacing the data of the population of Brazil and its federation units updated for the year 2020 [54]. All parameters that depended on populations were recalculated. Other columns were included in this tab with several other parameters of interest. The data that appears in Table 1 were taken from that last tab.

All graphs were made in the Excel File S3 of 28 tabs. The order and the names of the tabs follow the order and abbreviations of the states and the federal district as shown in Table 1. In each tab, the columns from C to T, starting from row 7, were copied from the File S2, from column T to AK and from the rows between the start date of 1 March 2020 and the end date of 15 March 2021. It is worth observing that a technical difficulty was found in formatting the dates contained in the primary data spreadsheet, therefore, the dates that appear in column B of all tabs of File S3 were placed independently: the initial date of 1 March 2020 of cell B7 was placed manually; subsequent dates from cell B8 were obtained by adding 1 day to the previous day, for example, B8 = B7 + 1, until reaching the row of the final date (15 March 2021). Thus, the dates of the Files S2 and S3 exactly match. The formatting of the horizontal axis (containing the dates) and the secondary vertical axis (indicating the deaths per day per million people), which appear in the final graphs shown in Figure 2, Figure 3, Figure 4, Figure 5 and Figure 6, were made separately.

## Appendix B. A Discussion about the Main Reasons for the Municipalities of Pará and Their Physician to Adhered to Early Ambulatory Treatment in the Public Healthcare System

To address this issue, five points are discussed: (1) Access of patients to the public healthcare system and/or to the private medical network; (2) Main approaches of the physicians to the first symptoms of COVID-19; (3) Need for a prescription for certain medicines, and availability of the medicines to private consumers; (4) Acquisition of medicines by the public sector; (5) Main causes for the municipalities and their physicians to adhere to early ambulatory treatment in the public healthcare system.

As already mentioned in Section 1, the states of Amazonas and Pará present similar SES distribution. The majority of patients have medium to low SES, and they can only have access to the public healthcare system, which is linked to the Brazilian SUS (Unified Health System). A smaller proportion of patients, primarily those with high SES, may have access to private physicians and/or to the private medical network, mainly through private healthcare plans (health insurance).

In general, two of the used approaches of the physicians to treat the first symptoms of COVID-19 are: (a) to make exams and treat the patients according to their symptoms because COVID-19 was a new disease and no medicine had been specifically approved by the control agencies (in the case of Brazil, ANVISA), and (b) to make exams and perform early ambulatory treatment according to medical protocols established early in the pandemic. It is outside the scope of the present work to discuss these protocols. Approach (a) usually involves the necessary medical exams, such as COVID-19 diagnostic test, if available. Usual medications are prescribed to treat the symptoms, such as an antipyretic in case of fever. The medicines in general can be purchased at pharmacies or, eventually, made available by the doctor or the healthcare unity that the patient is attended. Most patients do not progress to severe cases of the disease. However, if the patient’s health condition became worse, appropriate medical interventions have to be performed. Sometimes it is necessary to admit the patient to an intensive care unit in hospital where intubation procedures may be required. Approach (b) usually involves the necessary medical exams, such as COVID-19 diagnostic test, if available, but an early ambulatory treatment begins if the patient presents the first symptoms of the disease, according to medical protocols established early in the pandemic. It is informed that such a procedure is important since part of the tests to detect COVID-19 take several days for the result to be released, and many tests give false-negative results [8,12,13]. As mentioned in Section 1, the medicines that have been recommended in scientific studies [7,8,12,13,14] are: hydroxychloroquine, azithromycin and ivermectin, among others. It is worthy to mention that it is outside the scope of the present work to discuss first symptoms of the disease, and the doses, the effects, and the actions of the medicines, combined or not. Some critics of approach (b) usually say that that there is no evidence that the medicines have any benefit at all stages of COVID-19 [10,55], and the patients can suffer from side effects from the medications [55]. However, the researchers that support early ambulatory treatment say that it is not possible to know, a priori, how the disease will develop in any particular person, and it is especially important starting the treatment as soon as possible at the first symptoms [7,8,12,13,14], because this improve the combating of the disease; they say that the benefits that can be achieved (in terms of saving lives) outweigh the harm caused by possible side effects from the medicines.

Concerning the need for a prescription for the medicines, in the beginning of the pandemic, only azithromycin, because it is an antibiotic, needed a medical prescription in Brazil. Hydroxychloroquine and ivermectin were over-the-counter drugs that could be obtained without a prescription. However, shortly after the beginning of the pandemic, a prescription was necessary to purchase hydroxychloroquine after 20 March 2020 [56] and ivermectin after 22 July 2020 [57]. The need for a prescription for ivermectin was revoked on 1 September 2020 [58], but that for hydroxychloroquine is still maintained. With regard to the availability of medicines, shortly after the start of the pandemic, the press, and the social networks, mainly messaging apps such as WhatsApp, reported the possibility of using some medicines, such as those above mentioned, to combat COVID-19. It was also reported that some of the medicines might not work and that they could cause side effects. Even with the critical news, there was a great demand for these medicines in Brazil, and the price of two of them (hydroxychloroquine and ivermectin) increased substantially, and they were practically sold out in the pharmacies for some months. Therefore, the purchase of two of the medicine by patients individually was very restricted, even for those who had a prescription and had financial resources to purchase them. The purchase of these medicines by healthcare plan operators of the private medical network, such as Unimed Belém, was possible because the purchase was made directly from manufacturers or representatives.

Regardless of the pandemic, the purchase of medicines by the public healthcare system for distribution to patients is particularly important in Brazil, especially for low SES patients. In many cases, when public healthcare units do not provide the medications to the patients, even with a prescription and the medications available at pharmacies, many low SES patients have financial difficulties to purchase them. In Brazil, public organizations must make the purchase of medicines and other supplies through a bidding process, which requires time and work, in addition to financial resources. If there is urgency, as in the case of the beginning of the pandemic, the bidding process may be simplified, but the public organization lawyers must prepare and publish a document with grounded justifications for the procedure, as the municipality of Afuá-PA did on 13 May 2020 [43]. However, this is laborious and requires determination, in addition to depending on financial resources. The great difference of the state of Pará in relation to Amazonas and the other states of Brazil was that the state government made the purchase process in a centralized way for all municipalities in the state. The public healthcare system received the medications in their municipalities without having to follow the entire purchase process, which involve bureaucratic issues, publication of documents, search for suppliers, payment and, finally, the receipt of the medications.

In that situation of May 2021, with a high mortality rate, the physicians in the public healthcare system of the state of Pará were able to choose the approach of early ambulatory treatment after 21 May 2020, because: (1) the results of the other approach were not so successful (the number of deaths was increasing steadily); (2) the actions for COVID-19 of Unimed Belém and the municipality of Ourilândia do Norte-PA for their patient in the beginning of May 2020 [42,44] was an incentive; and (3) in Pará the medicines were available and provided free of charge by the state government. In the public medical healthcare units of Pará, the patients received the medicines directly at the end of the medical consultation; they did not need to go to the pharmacy. The particularly good practical result achieved from the fourth week of May 2020 in Pará gave credibility to the early ambulatory treatment in the public healthcare system.

In Amazonas, early ambulatory treatment was difficulted in the public healthcare system mainly because the state government did not support early ambulatory treatment, and, therefore, did not purchase the medicines.
